# A Peano-Gosper Fractal-Inspired Stretchable Electrode with Integrated Three-Directional Strain and Normal Pressure Sensing

**DOI:** 10.3390/nano15171370

**Published:** 2025-09-05

**Authors:** Chunge Wang, Yuanyuan Huang, Zixia Zhao, Haoyu Li, Chen Liu, Zhixin Jia, Yanping Wang, Qianqian Wang, Sheng Zhang

**Affiliations:** 1 Zhejiang Key Laboratory of Multiflow and Fluid Machinery, Zhejiang Sci-Tech University, Hangzhou 310018, China; 2023220504014@mails.zstu.edu.cn (Y.H.); wangyp@zstu.edu.cn (Y.W.); 2 School of Mechanical and Energy Engineering, NingboTech University, Ningbo 315100, China; wangchunge@nit.zju.edu.cn (C.W.); zhaozx0507@outlook.com (Z.Z.); lhy082705@outlook.com (H.L.); jzx@nit.zju.edu.cn (Z.J.); 3 Division of Engineering in Medicine, Department of Medicine, Harvard Medical School, Brigham and Women’s Hospital, Cambridge, MA 02139, USA; liuchen@nit.zju.edu.cn; 4 Ningbo Innovation Center, Zhejiang University, Ningbo 315100, China; 5 Faculty of Science and Engineering, University of Nottingham Ningbo, Ningbo 315100, China

**Keywords:** Peano-Gosper fractal curve, stretchable electrode, three-directional strain sensing, normal pressure detection, liquid metal (EGaIn), wearable electronics, multifunctional sensing

## Abstract

A novel stretchable flexible electrode capable of simultaneously detecting isotropic three-directional strain and normal pressure has been developed. Inspired by the recursive symmetry of the Peano-Gosper fractal, the electrode integrates liquid metal (EGaIn) microchannels within a PDMS matrix to achieve uniform strain distribution and mechanically robust conductive pathways under large deformation. Within a strain range of 0–60%, the electrode exhibits highly consistent three-directional responses, with resistance variation across axes kept below 4% and a gauge factor (*GF*) standard deviation of only 0.0252. The device demonstrates low hysteresis (minimum DH = 0.94%), good cyclic durability, and reliable electromechanical stability. For normal pressure sensing (0–20 kPa), it provides a linear response (*R*^2^ ≈ 0.99) with a moderate sensitivity of 0.198 kPa^−1^. Wearable tests on the wrist, finger, and fingertip confirm the electrode’s reliable operation in multidimensional mechanical monitoring. This integrated fractal–liquid metal design offers a promising route for multifunctional sensing in applications such as soft robotics, human–machine interaction, and wearable electronics.

## 1. Introduction

The rapid development of wearable electronics has facilitated extensive applications in health monitoring [1,2,3,4], motion tracking [5,6,7], soft robotics [8], and human–computer interaction [9,10]. As core components, flexible electrodes must maintain stable signal acquisition under complex dynamic deformations, requiring adaptability to multidirectional mechanical stimuli. In practical scenarios such as human joint motion [11,12,13] and human–computer interaction [14,15], wearable devices often need to synchronously perceive multidimensional strain or pressure while retaining favorable electromechanical response characteristics.

Current research efforts fall into two main categories. At the material level, composite conductive networks based on nanomaterials such as CNTs, graphene, and MXene have been designed to respond to strains from multiple directions [16,17,18]. However, these approaches generally remain limited to in-plane sensing and exhibit directional anisotropy. At the structural level, strategies such as kirigami cuts, wavy patterns, and fractal-inspired designs have improved mechanical stretchability and sensitivity [19,20,21]. Despite progress, most Electrodes still fail to deliver isotropic three-directional responses while also lacking normal pressure sensing capability and hindering their application in complex deformation environments.

To overcome these limitations, this study develops a stretchable electrode integrating Peano-Gosper fractal geometry [22,23] and liquid metal (EGaIn) microchannels. The self-similar symmetric hexagonal arrangement of the fractal structure [24] enables uniform stress dispersion and directional balance, providing the geometric foundation for isotropic three-directional response. Simultaneously, the high fluidity of EGaIn ensures reliable conductivity under tensile/compressive loads. This design achieves synchronous three-directional strain and normal pressure sensing within a single device without compromising mechanical integrity.

Experimental results validate the proposed architecture: the electrode maintains three-directional response consistency within 0–60% strain (coefficient of variation < 4%), demonstrates minimal hysteresis with robust cyclic stability, and exhibits a linear pressure response (*R*^2^ ≈ 0.99) with moderate sensitivity (0.198 kPa^−1^), fulfilling requirements for multidimensional mechanical sensing.

In summary, this work presents the first flexible electrode that integrates isotropic three-directional strain and normal pressure sensing on a unified platform. We focus on the development and fundamental characterization of this fractal-inspired design, rigorously demonstrating its core electromechanical properties—including isotropic multidirectional strain response, integrated pressure sensitivity, and robust cycling stability. This work establishes the device as a high-performance foundational sensing element, rather than a fully packaged sensor system, addressing critical limitations of existing devices and paving the way for next-generation wearable systems, soft robotics, and multimodal human–machine interfaces.

## 2. Experimental Section

### 2.1. Materials and Equipment

DuPont Film (SD 238, DuPont, Wilmington, DE, USA), Polydimethylsiloxane (PDMS) (Dow Corning Sylgard 184, base:curing agent = 15:1 by volume ratio), Eutectic gallium-indium alloy (EGaIn) (99% purity, Dingguan Metal Co., Dongguan, China), Ecoflex 00-30 (00-30, Smooth-On, Macungie, PA, USA), Single-core silver-plated copper wire (Φ 0.25 mm, Shanghai Xiangyu Wire & Cable Co., Shanghai, China), Foam tape (width 9 mm, Deli Group Co., Ningbo, China), Tempered glass plate (100 × 100 × 3 mm^3^), Mask plate (304 stainless steel, thickness 0.1 mm, Shenzhen Dongcheng Xin Metal Products Co., Dongguan, China), Syringe (5 mL capacity, Hunan Good Nurse Medical Equipment Co., Changsha, China), Anhydrous sodium carbonate (Sichuan Xilong Scientific Co., Chengdu, China).

Electronic balance (FA1004 model, Shanghai Qiqiang Instrument, Shanghai, China), Constant-temperature heating plate (V-1015 model, Weiteke Electronics Co., Shenzhen, China), Digital multimeter (Keysight Technologies, Wokingham, UK, model 34461A),Flexible electronics tester (FT2000 model, Shanghai Mipower Electronics Technology Co., Shanghai, China), Electric thermostatic blast drying oven (DMG-9070A model, Shanghai Jinghong Experimental Equipment Co., Shanghai, China), Tribological testing platform (Boruiyan Intelligent Technology Co., Ltd., Suzhou, China), UV exposure chamber (Shenzhen 922 Digital Technology Co., Shenzhen, China).

All reagents were used as received without further purification.

### 2.2. Electrode Fabrication

#### 2.2.1. Structural Design

Fractal structures offer novel approaches for optimizing the multidirectional stretchability of flexible electrodes due to their unique self-similarity and scale-invariance characteristics. From the perspective of fractal geometry theory, fractal curves with Hausdorff dimension [25] exceeding their topological dimension exhibit recursively iterative topological similarity across multiple spatial scales [26]. This geometric property enables uniform stress–strain field distribution at micro/nanoscale levels during macroscopic deformation. Mechanical simulation studies reveal that the recursive branching characteristics of fractal structures can effectively suppress stress concentration through multistage energy dissipation mechanisms, with strain energy density demonstrating self-similar distribution patterns during two-directional/three-directional stretching [27,28].

**Figure 1:**
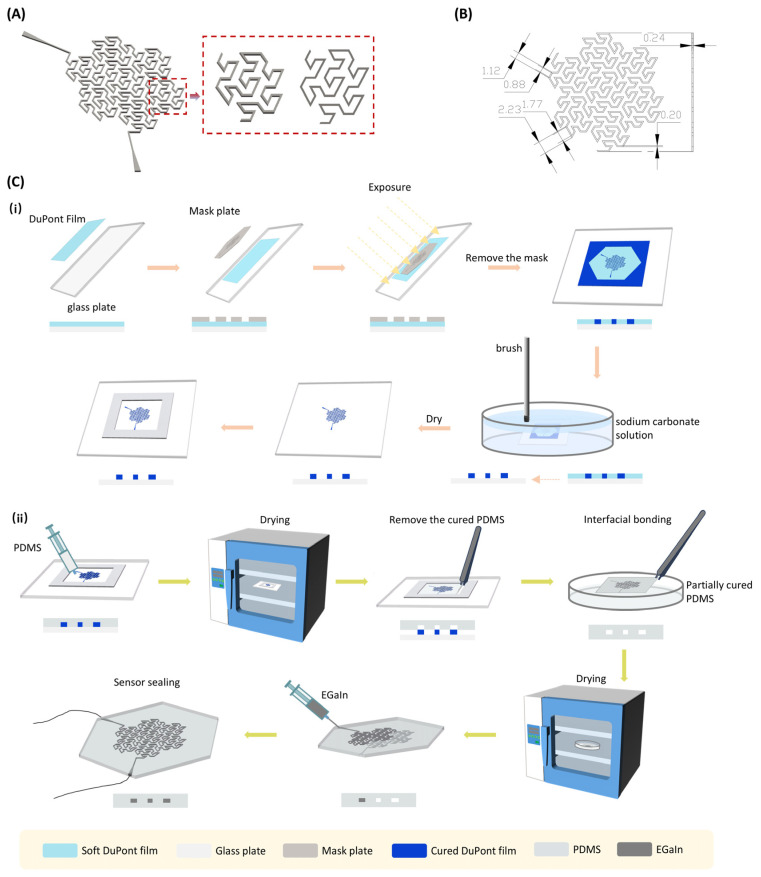
Fractal structural pattern design and electrode fabrication process. (**A**) Peano-Gosper fractal structure channel design. (**B**) Fundamental dimensions of the EGaIn (eutectic gallium-indium) infusion channel. (**C**) Electrode fabrication process: (**i**) Mold fabrication procedure, (**ii**) Electrode assembly procedure.

The Peano-Gosper fractal structure, generated through hexagonal iteration with a Hausdorff dimension of approximately 1.771, utilizes its high dimensionality to distribute stress via multilevel branching. Under tensile/shear deformation, localized strain propagates to adjacent domains through hierarchical nesting, thereby preventing strain concentration [24]. The self-similar tiling of hexagonal units endows the curve with highly symmetric strain response capabilities along three directions, establishing the theoretical foundation for isotropic mechanical responses. These distinctive mechanical properties render the Peano-Gosper fractal an ideal platform for fabricating flexible electrodes with low strain sensitivity, high uniformity, and three-directional isotropic response. Figure 1A illustrates the Peano-Gosper curve (iteration order *n* = 3) employed in the design, while Figure 1B details the channel dimensions with a width of 0.2 mm and height of 0.24 mm.

#### 2.2.2. Mold Fabrication

Soft lithography is commonly employed for fabricating fluidic channels in microfluidic chips, being well-suited for achieving channel widths and depths on the micron scale. This study utilized a modified soft lithography process to fabricate the mold required for injecting liquid metal into the electrode. The primary steps of the mold fabrication procedure are illustrated in Figure 1C(i).

**Figure 2:**
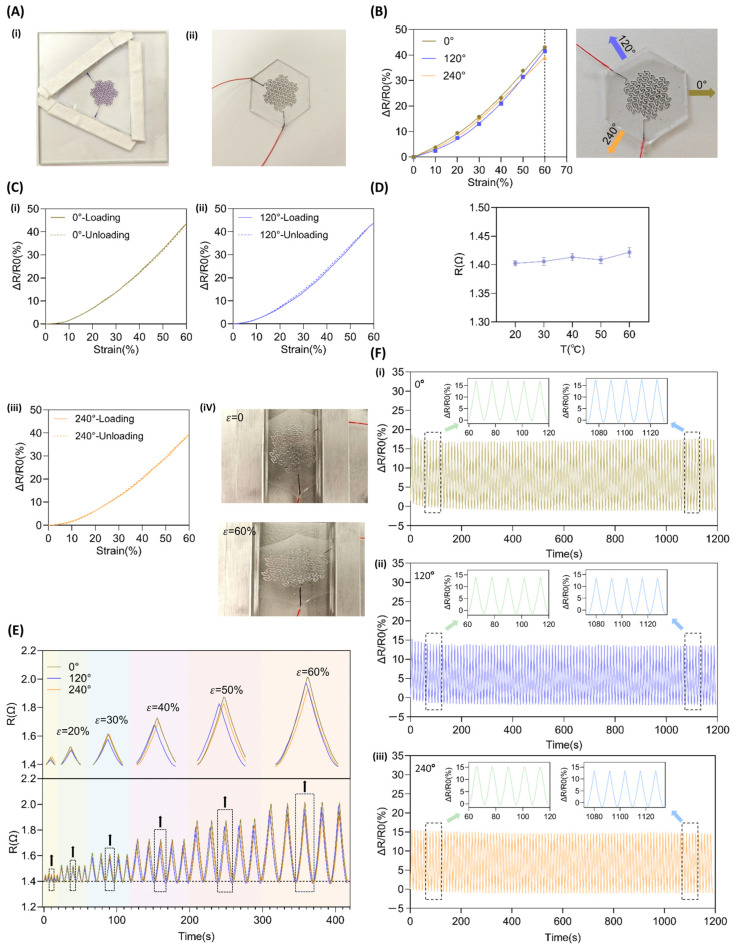
Electrode preparation results and basic electromechanical performance. (**A**) The completed mold and electrode: (**i**) physical image of the self-made mold, (**ii**) physical image of the finished electrode. (**B**) Relative resistance change rate of Direction 1 (0°), Direction 2 (120°), and Direction 3 (240°) under different strains. (**C**) Hysteresis loop of electrode loading-unloading under 60% strain condition: (**i**) Direction 1 (0°), (**ii**) Direction 2 (120°), (**iii**) Direction 3 (240°), (**iv**) physical images of the electrode’s initial state and 60% stretched state. (**D**) Resistance Variation Graph at Different Temperatures (**E**) Resistance change rate of the electrode under incremental strain (10–60%) in Direction 1 (0°), Direction 2 (120°), and Direction 3 (240°). (**F**) Resistance change rate of the electrode after 100 stretch-release cycles under three-directional 30% strain condition: (**i**) Direction 1 (0°), (**ii**) Direction 2 (120°), (**iii**) Direction 3 (240°).

First, a Peano-Gosper fractal pattern was designed using AutoCAD2024 and fabricated into a 304 stainless steel mask via laser cutting. To achieve the desired channel depth, multiple layers of DuPont film (with a single-layer thickness of approximately 40 μm) were laminated onto a glass substrate; specifically, six layers were laminated in this study to obtain channel structures with a depth of approximately 240 μm. A laminator was used during the application of each film layer to eliminate air bubbles and liquids trapped between layers, ensuring tight interlayer adhesion. Subsequently, the mask was aligned over the laminated region and secured with another glass plate to prevent displacement. The assembly was then transferred to an exposure chamber and exposed for 1 h under light-shielding conditions. After exposure, the protective layer on the DuPont film surface was peeled off. The film was then immersed in a 1 wt% Na_2_CO_3_ solution, and unexposed regions were gently washed away using a soft brush to form the channel structure. This developing process exploits the principle that unexposed regions dissolve, resulting in a microchannel with a negative pattern. Thereafter, the dried channel pattern was framed and secured using foam tape, forming a complete PDMS casting mold structure. A photograph of the final fabricated channel mold is shown in Figure 2A(i).

#### 2.2.3. Electrode Fabrication Process

Figure 1C(ii) illustrates the complete fabrication process of the flexible electrode, including PDMS casting, channel layer encapsulation, EGaIn injection, and electrode packaging. The specific steps are detailed as follows: Polydimethylsiloxane (PDMS), prepared by thoroughly mixing the base and curing agent at a 15:1 volume ratio, was injected into the aforementioned mold using a syringe. After 30 min of degassing, the PDMS was cured in an 80 °C forced-air oven for 30 min. The cured PDMS was then demolded and trimmed to the appropriate dimensions. Subsequently, another portion of PDMS was poured into a Petri dish, subjected to vacuum degassing, and heated to a partially cured state. The previously obtained channel layer was gently pressed onto the surface of this partially cured PDMS layer. The assembly was then returned to the 80 °C oven to complete the bonding and curing process, thereby forming an encapsulated channel structure. This interlayer encapsulation enhances overall flexibility and sealing while providing stable support for subsequent EGaIn injection. Thereafter, a syringe was used to inject clean eutectic gallium-indium alloy (EGaIn) into the microchannels, forming a continuous liquid conductive network. To establish electrical connections, silver-plated copper wires were inserted into both ends of the channels. The wire-channel interfaces were then encapsulated by applying Ecoflex, which not only secures the wires and prevents leakage of the liquid metal, but also provides a hermetic moisture barrier to protect the conductive pathways from humidity-induced degradation.

The PDMS layer structure, EGaIn filling method, and interface encapsulation steps involved in this fabrication process are all annotated in Figure 1C(ii), ensuring consistency between the structural design and process implementation. A photograph of the final fabricated electrode is shown in Figure 2A(ii), with an overall side length of approximately 20 mm, a height of about 35 mm, and internal microchannel dimensions of 0.2 mm in width and 0.24 mm in height.

### 2.3. Electrode Testing and Characterization

To characterize the in-plane multidirectional stretchability of the electrode, we conducted tensile testing under uniaxial strain applied along three distinct directions using a flexible electronics testing system: Direction 1 (0°), Direction 2 (120°), and Direction 3 (240°), with directional definitions as shown in Figure 2B. All tests were performed at a strain rate of 1 mm/s with an effective initial gauge length of 20 mm between fixtures, covering a strain range of 0–60%. To evaluate its thermal stability, the electrode was heated using a Constant-temperature heating plate with measurements taken at 10 °C intervals from ambient temperature to 60 °C; resistance values were recorded after reaching thermal equilibrium at each temperature step under zero applied strain. Concurrently, 100 consecutive stretch-release cyclic tests were implemented for each direction at a fixed strain level of 30%. For normal pressure sensitivity characterization, the electrode was mounted on the load cell module of a tribological testing platform, which was equipped with a Gamma six-axis force/torque sensor (ATI Industrial Automation, Apex, NC, USA). The system was ensured to be free from external mechanical interference prior to testing. A flat, rigid indenter was used to ensure uniform pressure distribution over the entire active sensing area (9 cm^2^) of the electrode. The normal force was applied vertically downward and increased in increments of 1 N, with the corresponding resistance values recorded at each step. To evaluate practical performance, the electrode was affixed to three anatomical locations (fingertip, finger joint, and wrist), with resistance changes recorded in real-time during corresponding motions.

The two key parameters of flexible force sensors are the gauge factor (*GF*) and sensitivity (*S*). The gauge factor *GF* is the ratio of relative resistance change to relative strain change [29].

(1) $$GF=\frac{\left(\Delta R∕R_{0}\right)}{\left(\Delta L∕L_{0}\right)}=\frac{\left(R_{1}-R_{0}\right)∕R_{0}}{\left(\Delta L∕L_{0}\right)}$$

where Δ*R* is the change in resistance, Δ*L* is the change in length, *R*_0_ is the initial resistance without applied strain, *L*_0_ is the initial length, and *R*_1_ is the recorded resistance after stretching.

Sensitivity *S* is the ratio of relative resistance change to pressure change.

(2) $$S=\frac{\left(\Delta R∕R_{0}\right)}{P}=\frac{\left(R_{1}-R_{0}\right)∕R_{0}}{P}$$

where *P* is the applied pressure, *R*_0_ is the initial resistance without any pressure applied, and *R*_1_ is the recorded resistance after a certain pressure is applied.

The hysteresis phenomenon in flexible sensors refers to the difference in sensor output when the same strain is applied during loading and unloading. Reducing hysteresis is crucial for obtaining reliable and accurate measurements in real-time applications. A lower degree of hysteresis (DH) indicates less lag in the electrical response [30].

(3) $$\mathrm{DH}=\frac{A_{\mathrm{Loading}}-A_{\mathrm{Unloading}}}{A_{\mathrm{Loading}}}\times 100\%$$

where *A_Loading_* and *A_Unloading_* are the areas of the loading and unloading curves, respectively.

In addition to DH, the Hysteresis Error (*Hyst_err_*)at a certain strain value can be calculated by the resistance difference between loading and unloading. The Hysteresis Error (*Hyst_err_*) quantifies the maximum deviation between the loading and unloading curves at a given strain value, expressed as a percentage of the full-scale output. This parameter is critical for assessing the accuracy and repeatability of flexible sensors under cyclic deformation [31].

(4) $${\mathrm{Hyst}}_{\mathrm{err}}=\frac{r_{0}}{u_{0}}\times 100\%$$

where *r*_0_ is the maximum difference in relative resistance change between the loading and unloading parts of the hysteresis cycle, and *u*_0_ is the maximum resistance change at full scale.

## 3. Results and Discussion

### 3.1. Electromechanical Stability

The relative change in resistance of flexible electrodes under stress is a key indicator for evaluating their strain response performance and stability, especially for multi-directional stretchable electrodes. The consistency of response under stretching in various directions can directly verify their multi-directional adaptability and isotropy level. As shown in Figure 2B, within the strain range of 0–60%, the difference in resistance change rates under stretching along the three directions of the electrode is less than 4%, with the Direction 1 response slightly higher than Direction 2 and Direction 3. This result indicates that the Peano-Gosper fractal structure can effectively achieve symmetric strain distribution during macroscopic stretching, endowing the electrode with excellent three-directional consistency.

To further quantitatively analyze the similarity of the responses of each direction, linear fitting was performed on the data in Figure 2B, and the R^2^ values for Direction 1, Direction 2, and Direction 3 were obtained as 0.9712, 0.9488, and 0.9740, respectively, indicating a good linear relationship between the relative resistance change and strain. The corresponding gauge factors (*GF*) were 0.72, 0.70, and 0.66, with a standard deviation of only 0.0252, indicating extremely low dispersion, further confirming the consistency of the electrode’s anisotropic responses. In comparison, the waveform-structured liquid metal flexible electrode in reference [20] exhibited a higher *GF* (with a maximum value of 2.67), but it only had uniaxial tensile capability, and the response curve was significantly anisotropic. The MXene wrinkled membrane structure in reference [18] also only achieved biaxial detection, with a strain response difference of 9.6% between the two directions. Although the *GF* of the electrode in this study is at a moderate level (0.66–0.72), it achieved a response difference of less than 4% under three-directional strain, establishing a stable and balanced three-directional isotropic response performance, with significant advantages in structural functional integration and practicality. The reason for the moderate gauge factor but outstanding stability of this electrode mainly lies in the stress regulation mechanism of the fractal structure: the Peano-Gosper structure uniformly disperses strain across multiple hierarchical branches, avoiding stress concentration, thereby resulting in a more gradual change in resistance during stretching. While this moderates the resistance response to stretching, it significantly improves mechanical robustness and sensing reliability. Therefore, compared to waveform or kirigami structures, this electrode exhibits a more suitable performance balance for multi-directional dynamic detection in complex environments.

Figure 2C shows the loading-unloading hysteresis loops of the three directions under 60% maximum strain. After calculation, the hysteresis degrees (DH) of Direction 1, Direction 2, and Direction 3 are 0.94%, 2.76%, and 1.17%, respectively; the corresponding hysteresis errors (*Hyst_err_*) are 1.41%, 2.41%, and 2.03%. All three directions exhibit a low hysteresis level, indicating that during the cyclic stretching-release process, the electrode’s resistance response is fast, repeatable, with low energy dissipation, and demonstrates excellent dynamic consistency and signal recovery capability. This low hysteresis characteristic is mainly attributed to: (1) the fluidity of the liquid metal EGaIn, which can adaptively reconstruct conductive paths under strain, avoiding the accumulation of irreversible deformation; (2) the uniform stress diffusion effect brought by the fractal structure reduces the probability of interface slip or structural damage; (3) the elastic modulus of the PDMS matrix matches the structural encapsulation, making the stress–strain response more stable.

To evaluate dynamic stability, Figure 2F shows the results of 100 cycles of stretching in Direction 1, Direction 2, and Direction 3 under 30% strain. The initial response showed slight fluctuations but rapidly stabilized. The resistance changes in each direction were controlled within a range of 0–17%, with consistent curve trends and no response drift or curve distortion. These results further support the performance advantages reflected in the aforementioned *GF* and hysteresis data, demonstrating that the fractal–liquid metal system exhibits high reliability under multi-directional dynamic loading.

As shown in Table 1, this table compares the tensile performance of our electrode with the flexible electrodes that have been developed so far. In the table, the flexible electrode with the Peano-Gosper fractal curve structure in this study has lower DH and *Hyst_err_* compared to the other two flexible sensors (waveform structure [20] and third-order Peano structure [21], both using Ecoflex 00-30 as the substrate, with tensile strain test ranges of 0–320% and 0–100%, respectively). Moreover, the flexible electrode in this study can perform three-directional stretching, showing similar performance in all three directions. However, compared to the non-fractal waveform structure, both this work and the third-order Peano fractal structure exhibit lower *GF*, which is consistent with the previous explanation.

To further verify the stability under multidirectional stretching of the Peano–Gosper fractal structure, the resistance change curves of the electrode under cyclic loading–unloading along three directions—at 10%, 20%, 30%, 40%, 50%, and 60% strain—were tested using a flexible electronic material testing system, as shown in Figure 2E. During stretching, minor differences in resistance were observed among the three directions, which may be attributed to systematic deviations such as minor inconsistencies in the encapsulation junctions. For instance, additional stress from the fixture on the wire interface during stretching may introduce slight resistance variations across directions. However, the overall difference remained below 0.105 Ω and did not affect the general performance assessment. The trends in resistance change were highly consistent across all three directions, with no divergence in response as strain increased. These results confirm that the electrode structure maintains strong strain uniformity and directional stability even under large deformation, demonstrating high robustness and further supporting the feasibility of using the Peano–Gosper fractal curve to achieve multidirectional stretchability.

To ensure the reliability and thermal stability of flexible electronic devices in practical applications such as medical monitoring, electronic skin, and wearable devices, characterizing their temperature-dependent performance is essential. Temperature fluctuations may cause signal drift or functional failure. Therefore, this study evaluated the thermal stability of multidirectional stretchable electrodes. Under external force-free test conditions, the results indicated that within the normal operating temperature range of the device (20 °C to 60 °C), the resistance variation remained minimal—stabilizing around 1.41 Ω (as shown in Figure 2D) with a fluctuation rate of only approximately 0.537%. These results demonstrate that the electrode exhibits excellent thermal stability, making it suitable for most practical application scenarios.

In summary, the electrode achieved a resistance response difference of less than 4% across three directions within the strain range of 0–60%, with a *GF* standard deviation of 0.0252. It demonstrated excellent cyclic stability and low hysteresis error, fully validating the effectiveness of the Peano–Gosper fractal structure in constructing an isotropic and stable strain sensing platform. Furthermore, the device exhibited remarkable thermal stability, with merely 0.518% resistance fluctuation over a practical temperature range of 20–60 °C, ensuring reliable operation in varying environmental conditions. While ensuring force–electric consistency, the electrode also exhibits strong environmental adaptability, making it a highly capable solution for complex multidirectional dynamic sensing scenarios.

### 3.2. Cyclic Strain Stability

The stability of flexible electrodes during long-term and repeated use is a critical indicator for meeting the practical application requirements of wearable devices. Frequent deformation may lead to material fatigue, interface aging, or degradation of conductive pathways, resulting in signal drift and performance decay. To evaluate the cyclic stability of the stretchable flexible electrode developed in this study, 100 cycles of tensile testing at 30% strain were applied along each of the three in-plane directions. The test results are presented in Figure 2F.

During the initial testing phase, slight fluctuations in the rate of resistance change were observed due to the early-stage adaptation of materials and structures. However, from the 10th cycle onward, the response rapidly stabilized, with the resistance change rates in all three directions remaining within stable ranges: Direction 1 fluctuating between 0–17%, Direction 2 between 0–15%, and Direction 3 between 0–16%. Throughout the 100 loading–unloading cycles, no systematic drift occurred in the response curves, and the peak values showed no significant increase or attenuation, indicating that the conductive pathway structure sustained no cumulative damage.

In addition, after each cycle of unloading (strain returning to zero), the electrode resistance can quickly recover to near the initial value, with no baseline drift, demonstrating good strain recovery and structural rebound characteristics. The highly consistent morphology of the cycle curves in the figure indicates that the fluidity of EGaIn and the stress homogenization characteristics of the fractal structure together ensure long-term stable force-electric response.

This experiment selected 100 cycles as the test period, primarily based on the following two considerations: firstly, existing research indicates that the performance changes of flexible strain electrodes are most significant during the initial few dozen cycles [30]; secondly, the electrode has already demonstrated a stable response trend within 100 cycles, with subsequent changes tending to saturate. It is important to note that the cycling tests in this study were limited to approximately 200 cycles, a constraint primarily influenced by material aging considerations of the PDMS matrix and the long-term interconnection reliability between the liquid metal channels and the silver-plated copper wires under prolonged mechanical agitation. Although this test constitutes a medium to short-term evaluation, it can still effectively reflect the repeated-use stability of the device in practical wearable application scenarios (such as daily finger or wrist activities). In subsequent work, the flexible electrode can be further optimized and extended to longer cycling tests (such as 500 or 1000 cycles) to verify its long-term reliability.

This result echoes the consistency of the three-directional response observed in the unilateral tensile test in Section 3.1, further validating the coordinated deformation capability and conductive path stability of the Peano–Gosper fractal structure under dynamic multidirectional strain conditions.

### 3.3. Pressure Sensitivity

**Figure 3:**
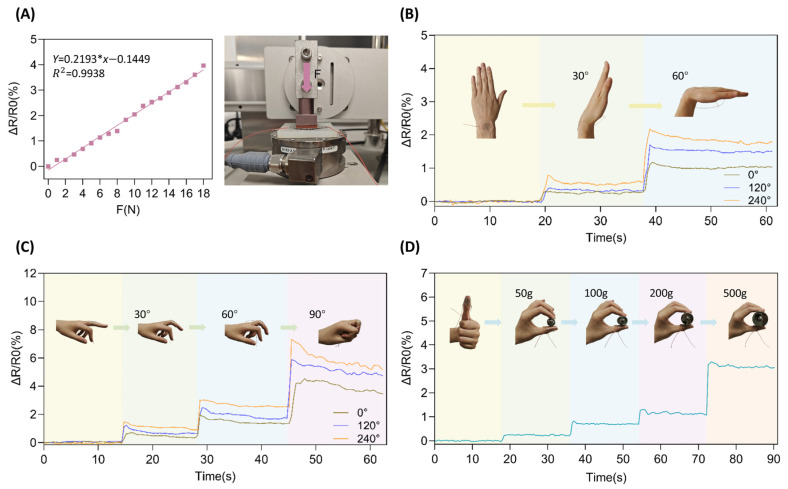
Normal force testing of the electrode and wearable applications. (**A**) Resistance change rate during normal pressure testing of the electrode. (**B**) Wrist bending test. (**C**) Finger bending test. (**D**) Fingertip pressure test.

To verify whether this electrode has effective normal pressure sensing capability in addition to in-plane tensile response, this paper conducted a normal loading test on it using a friction test platform. The test area was 9 cm^2^, corresponding to a pressure range of 0–20 kPa. To ensure accuracy, the pressure sensing module was calibrated and verified to be free from external influences prior to each test. Figure 3A shows the relationship between the relative resistance change rate and the applied pressure, as well as the physical test image. The experimental results indicate that the electrode output signal has a good linear relationship with pressure changes, with a coefficient of determination *R*^2^ ≈ 0.99 for the fitted curve, and a sensitivity of *S* = 0.198 kPa^−1^ within the test range.

This sensitivity level is significantly higher than most pure strain sensors without normal pressure sensing capability (generally *ΔR/R*_0_ response to pressure < 0.05%/kPa) [32,33], enabling stable detection of common touch pressure and slight grip force; however, this sensitivity is still lower than structured sensors specifically designed for high-sensitivity piezoresistive response (such as microstructured corrugated or porous interface devices, with typical sensitivity reaching above 0.5–1.5 kPa^−1^) [34]. The electrode functions as a strain-sensing element with pressure enhancement, achieving dual-mode detection without structural complexity.

The comprehensive test results indicate that this electrode has successfully overcome the limitations of traditional multidirectional strain electrodes in terms of weak normal perception, demonstrating characteristics such as high linearity, wide range, and moderate sensitivity. It has the potential for applications in scenarios such as lightweight force feedback and human–machine interaction recognition.

The test response process shown in Figure 3D indicates that as the applied pressure gradually increases from 50 g (approximately 0.5 kPa) to 500 g (approximately 5.5 kPa), the relative resistance change rate increases from 0.25% to 3.1%, with the overall response being stable and showing no significant drift or hysteresis. This demonstrates that the designed fractal–liquid metal structure exhibits sensitivity to multiple mechanical stimuli, enabling simultaneous detection of both strain and pressure. This multimodal sensing capability is integrated into a single device, laying the groundwork for subsequent signal decoupling through algorithmic or circuit design.

In addition, the wearable demonstration tests in Figure 3B–D further confirm the effectiveness of the pressure response of this electrode in actual grasping behaviors. Whether it is finger bending or light fingertip pressure, the resistance changes under different loads show continuity and distinguishability, reflecting the adaptability and practical stability of this structure to complex biological force fields.

## 4. Application Testing of Flexible Electrodes in Wearable Sensing

As a core component of flexible wearable sensors, multidirectional stretchable electrodes demand critical performance validation. To evaluate the developed multidirectional stretchable electrode under simulated multidimensional deformation environments, medical-grade adhesive film was utilized for direct mounting on human skin or joints during motion for in situ testing. The assessment focused on two core capabilities: (1) cooperative multidirectional strain perception (joint flexion) and (2) normal pressure detection (fingertip grasping), aiming to demonstrate its superior performance and practical utility in resolving the bottleneck issues of significant anisotropy and lack of normal response prevalent in conventional sensors.

### 4.1. Joint Motion Monitoring

To verify the application capability of the prepared multidirectional stretchable flexible electrode in practical strain monitoring scenarios, the electrode was first attached to the wrist. The mounting orientation of the electrode was altered to test the corresponding resistance changes when the wrist was bent to 30° and 60°, respectively (tests were conducted with the electrode attached along Direction 1, Direction 2, and Direction 3, with data for each direction collected from independent bending experiments). As the skin surface stretches during wrist bending, the electrode exhibits resistance changes in response to applied stress. By analyzing the resistance change curves of the electrode, the degree of wrist bending can be effectively monitored.

As shown in Figure 3B, when the wrist was bent to 30°, the resistance change rates along Direction 1, Direction 2, and Direction 3 were approximately 0.25%, 0.35%, and 0.50%, respectively. When bent to 60°, the resistance change rates of the three axes increase to approximately 1.0%, 1.5%, and 1.8%, respectively. Although there are some differences in the responses of the three axes, which may be due to factors such as the encapsulation joint, the fitting angle, and the joint motion trajectory, the overall trend shows that the resistance change increases with the bending angle, indicating that the electrode can effectively detect human movements of different amplitudes in multiple axes.

Furthermore, the electrode was mounted on the proximal phalanx of the finger, and its mounting orientation was altered to test the strain response performance to multi-angle finger flexion (data collection method consistent with the wrist test). As shown in Figure 3C, when the finger was bent to 30°, 60°, and 90°, the resistance change rates along Direction 1, Direction 2, and Direction 3 were approximately 0.5%, 0.7%, and 1.1%; 1.4%, 1.7%, and 2.5%; and 3.6%, 4.9%, and 5.4%, respectively. The variations observed among the three directions are consistent with the wrist test results mentioned earlier; however, the overall trend remains clear and the response stable, enabling accurate detection of strain variations induced by different degrees of finger bending.

The results indicate that the electrode can adapt to the monitoring needs of human joint movements in multiple axes, demonstrating excellent directional sensitivity and angular resolution, providing foundational support for multidimensional motion perception.

### 4.2. Fingertip Normal Pressure Monitoring

To explore the pressure sensing application of the electrode, the electrode was attached to the fingertip, and objects were gently picked up with two fingers. The weight change of the object was determined based on the change in resistance. As shown in Figure 3D, the image depicts the rate of resistance change of the electrode when picking up weights of different masses. When picking up a 50 g weight, the rate of resistance change was approximately 0.25%; for a 100 g weight, it was about 0.7%; for a 200 g weight, it was around 1.3%; and for a 500 g weight, it was approximately 3.1%. The test results indicate that the rate of resistance change increases with the increase in pressure (*R*^2^ ≈ 0.99), which is highly consistent with the results in Section 3.3, demonstrating its ability to detect different levels of daily grasping forces. Additionally, the figure shows that the electrode’s resistance change remained stable during the process of picking up weights, demonstrating its capability to stably monitor daily pressure.

The wearable application experiment verified that the multidirectional stretchable flexible electrode based on the Peano-Gosper fractal structure can simultaneously monitor multi-directional in-plane strain and normal pressure through a single device. Its three-directional isotropy, low hysteresis, and cyclic stability ensure the multidirectionality and accuracy of dynamic monitoring, providing technical support for complex human–computer interaction and health monitoring.

## 5. Conclusions

This work presents a stretchable flexible electrode that achieves, for the first time, integrated isotropic three-directional strain sensing and normal pressure detection within a single device. The electrode combines the self-similar and symmetric geometry of the Peano–Gosper fractal with the fluidic conductivity of liquid metal (EGaIn), enabling uniform strain distribution and robust signal stability under multidimensional deformation.

Experimental validation demonstrated excellent three-directional consistency within 0–60% strain, with resistance variation across directions below 4% and a gauge factor standard deviation of 0.0252. The device also exhibited low hysteresis (minimum DH = 0.94%), high cyclic durability, and reliable pressure response with linearity (*R*^2^ ≈ 0.99) and moderate sensitivity (0.198 kPa^−1^) under 0–20 kPa loading. Furthermore, the electrode showed remarkable thermal stability, with only approximately 0.537% resistance fluctuation over the practical temperature range of 20–60 °C, ensuring reliable operation in varying environmental conditions.

Compared to conventional uniaxial waveform designs [20], and earlier Peano-type structures [21], this approach enables simultaneous in-plane and out-of-plane mechanical detection without compromising integration or mechanical robustness. Wearable demonstrations involving wrist bending, finger flexion, and fingertip grasping further confirmed the electrode’s applicability to real-world human motion and force monitoring.

Overall, the Peano-Gosper fractal-inspired architecture offers a promising platform for the development of multifunctional wearable electrodes. Although the current sensitivity is moderate, its exceptional mechanical robustness, isotropic response, and integrated sensing capability are particularly advantageous for applications that prioritize reliability under large multi-axial deformations over high-resolution detection. These include proprioceptive feedback and tactile sensing in soft robotics, multidimensional motion tracking and rehabilitation assessment in healthcare, gesture recognition and force feedback in human–machine interfaces, as well as operator status and grip force monitoring in industrial safety. Future efforts to enhance the gauge factor—such as optimizing the microchannel geometry or incorporating hybrid conductive nanomaterials—could further broaden its applicability in scenarios requiring higher sensitivity. Additionally, although the electrode exhibits highly consistent responses under three-directional strain, its continuous and integrated liquid metal microchannel structure outputs a single resistance signal, making it unable to directly distinguish the specific direction of strain sources. The current design is more suitable for applications that require detecting the presence or magnitude of multidirectional strain but do not demand high directional discrimination. In the future, direction identification could be achieved by designing multi-channel independent sensing units or combining machine learning algorithms to decouple composite signals. Integration with wireless modules and data-driven algorithms will solidify its utility in smart healthcare, ambient intelligence, and next-generation robotic systems.

## Figures and Tables

**Table 1:** Comparison of key performance parameters between the Peano-Gosper structure electrode and flexible sensors in the referenced literature.

Channel Shape	Maximum *GF*	Minimum DH	Minimum *Hyst_err_*	Sensing Direction	Reference
Peano-Gosper	0.72	0.94%	1.41%	Three-directional	This work
Wave	2.67	1.02%	9%	Unidirectional	[20]
Peano II	0.84	3.70%	3.11%	Bidirectional	[21]

Note: Performance comparisons are contextual due to differing testing protocols (e.g., strain range, cycles) and primary device functions. References [20,21] are optimized for uniaxial/biaxial sensing, whereas this work prioritizes integrated three-directional isotropy and pressure detection, resulting in a distinct performance trade-off.

## Data Availability

All data needed to evaluate the conclusions in the paper are presented in the paper.

## References

[1] Ning C., Cheng R., Jiang Y., Sheng F., Yi J., Shen S., Zhang Y., Peng X., Dong K., Wang Z.L. (2022). Helical fiber strain sensors based on triboelectric nanogenerators for self-powered human respiratory monitoring. ACS Nano.

[2] Gao J., Fan Y., Zhang Q., Luo L., Hu X., Li Y., Song J., Jiang H., Gao X., Zheng L. (2022). Ultra-robust and extensible fibrous mechanical sensors for wearable smart healthcare. Adv. Mater..

[3] Zhang S., Zeng J., Wang C., Feng L., Song Z., Zhao W., Wang Q., Liu C. (2021). The application of wearable glucose sensors in point-of-care testing. Front. Bioeng. Biotechnol..

[4] Zhao W., Liu C., Wang Y., Li K., He Z., Zhou S., Zeng J., Ibrahim O.O., Zhang S., Wang Q. (2025). A flexible substrate-free electrochemical tattoo sensor with alcohol transfer printing method for real-time monitoring of sodium ions in sweat. Sens. Actuators B Chem..

[5] Hou C., Xu Z., Qiu W., Wu R., Wang Y., Xu Q., Liu X.Y., Guo W. (2019). A biodegradable and stretchable protein-based sensor as artificial electronic skin for human motion detection. Small.

[6] Shajari S., Ramakrishnan S., Karan K., Sudak L.J., Sundararaj U. (2022). Ultrasensitive wearable sensor with novel hybrid structures of silver nanowires and carbon nanotubes in fluoroelastomer: Multi-directional sensing for human health monitoring and stretchable electronics. Appl. Mater. Today.

[7] Wang C., Liu C., Shang F., Niu S., Ke L., Zhang N., Ma B., Li R., Sun X., Zhang S. (2023). Tactile sensing technology in bionic skin: A review. Biosens. Bioelectron..

[8] Wang Q., Sun X., Liu C., Wang C., Zhao W., Zhu Z., Ma S., Zhang S. (2023). Current development of stretchable self-powered technology based on nanomaterials toward wearable biosensors in biomedical applications. Front. Bioeng. Biotechnol..

[9] Maurya D., Khaleghian S., Sriramdas R., Kumar P., Kishore R.A., Kang M.G., Kumar V., Song H.-C., Lee S.-Y., Yan Y. (2020). 3D printed graphene-based self-powered strain sensors for smart tires in autonomous vehicles. Nat. Commun..

[10] Liu L., Xu W., Ni Y., Xu Z., Cui B., Liu J., Wei H., Xu W. (2022). Stretchable neuromorphic transistor that combines multisensing and information processing for epidermal gesture recognition. ACS Nano.

[11] Zhang H., Liu D., Lee J.-H., Chen H., Kim E., Shen X., Zheng Q., Yang J., Kim J.-K. (2021). Anisotropic, wrinkled, and crack-bridging structure for ultrasensitive, highly selective multidirectional strain sensors. Nano-Micro Lett..

[12] Yang C., Hou X., Zhang L. (2024). Microfluidics-derived microfibers in flexible bioelectronics. Mater. Futures.

[13] Bai J., Gu W., Bai Y., Li Y., Yang L., Fu L., Li S., Li T., Zhang T. (2023). Multifunctional flexible sensor based on PU-TA@ MXene janus architecture for selective direction recognition. Adv. Mater..

[14] Xiao W., Cai X., Jadoon A., Zhou Y., Guo Q., Tang J., Ma X., Wang W., Cai J. (2024). High-Performance Graphene Flexible Sensors for Pulse Monitoring and Human–Machine Interaction. ACS Appl. Mater. Interfaces.

[15] Dong H., Sun J., Liu X., Jiang X., Lu S. (2022). Highly sensitive and stretchable MXene/CNTs/TPU composite strain sensor with bilayer conductive structure for human motion detection. ACS Appl. Mater. Interfaces.

[16] He J., Feng J., Huang B., Duan W., Chen Z., Huang J., Li B., Zhou Z., Zeng Z., Gui X. (2024). Multi-directional strain sensor based on carbon nanotube array for human motion monitoring and gesture recognition. Carbon.

[17] Lu Z., Wang J., He L., Song J., Yang Z., Hammad F.A. (2024). High-performance multidirectional flexible strain sensor for human motion and health monitoring. ACS Appl. Mater. Interfaces.

[18] Yang R., Song H., Zhou Z., Yang S., Tang X., He J., Liu S., Zeng Z., Yang B.-R., Gui X. (2023). Ultra-sensitive, multi-directional flexible strain sensors based on an MXene film with periodic wrinkles. ACS Appl. Mater. Interfaces.

[19] Noushin T., Hossain N.I., Tabassum S. (2022). Kirigami-patterned highly stable and strain insensitive sweat pH and temperature sensors for long-term wearable applications. Proceedings of the 2022 IEEE Healthcare Innovations and Point of Care Technologies (HI-POCT).

[20] Chen J., Zhang J., Luo Z., Zhang J., Li L., Su Y., Gao X., Li Y., Tang W., Cao C. (2020). Superelastic, sensitive, and low hysteresis flexible strain sensor based on wave-patterned liquid metal for human activity monitoring. ACS Appl. Mater. Interfaces.

[21] Luo Y., Fan H., Lai X., Zeng Z., Lan X., Lin P., Tang L., Wang W., Chen Y., Tang Y. (2024). Flexible liquid metal-based microfluidic strain sensors with fractal-designed microchannels for monitoring human motion and physiological signals. Biosens. Bioelectron..

[22] Mandelbrot B.B. (1989). Fractal geometry: What is it, and what does it do?. Proc. R. Soc. Lond. A Math. Phys. Sci..

[23] Mandelbrot B.B., Aizenman M. (1979). Fractals: Form, chance, and dimension. Phys. Today.

[24] Carpinteri A., Chiaia B., Cornetti P. (2001). Static-kinematic duality and the principle of virtual work in the mechanics of fractal media. Comput. Methods Appl. Mech. Eng..

[25] Han S., Peng H., Sun Q., Venkatesh S., Chung K., Lau S.C., Zhou Y., Roy V.A.L. (2017). An overview of the development of flexible sensors. Adv. Mater..

[26] Mandelbrot B. (1967). How long is the coast of Britain? Statistical self-similarity and fractional dimension. Science.

[27] Falconer K. (2013). Fractal Geometry: Mathematical Foundations and Applications.

[28] Deane A.E., Keefe L.R. (1988). Multifractal spectra in homogeneous shear flow. Studying Turbulence Using Numerical Simulation Databases, 2. Proceedings of the 1988 Summer Program.

[29] Yu R., Xia T., Wu B., Yuan J., Ma L., Cheng G.J., Liu F. (2020). Highly sensitive flexible piezoresistive sensor with 3D conductive network. ACS Appl. Mater. Interfaces.

[30] Choi D.Y., Kim M.H., Oh Y.S., Jung S.-H., Jung J.H., Sung H.J., Lee H.W., Lee H.M. (2017). Highly stretchable, hysteresis-free ionic liquid-based strain sensor for precise human motion monitoring. ACS Appl. Mater. Interfaces.

[31] Di Tocco J., Presti D.L., Rainer A., Schena E., Massaroni C. (2022). Silicone-textile composite resistive strain sensors for human motion-related parameters. Sensors.

[32] Wang Y., Qin W., Yang M., Tian Z., Guo W., Sun J., Zhou X., Fei B., An B., Sun R. (2023). High linearity, low hysteresis Ti3C2Tx MXene/AgNW/liquid metal self-healing strain sensor modulated by dynamic disulfide and hydrogen bonds. Adv. Funct. Mater..

[33] Xu Y., Chen M., Yu S., Zhou H. (2023). High-performance flexible strain sensors based on silver film wrinkles modulated by liquid PDMS substrates. RSC Adv..

[34] Chang K., Guo M., Pu L., Dong J., Li L., Ma P., Huang Y., Liu T. (2023). Wearable nanofibrous tactile sensors with fast response and wireless communication. Chem. Eng. J..

